# Modular Synthesis of Trifunctional Peptide-oligonucleotide Conjugates *via* Native Chemical Ligation

**DOI:** 10.3389/fchem.2021.627329

**Published:** 2021-03-02

**Authors:** Mohadeseh Dastpeyman, John A. Karas, Azin Amin, Bradley J. Turner, Fazel Shabanpoor

**Affiliations:** ^1^ The Florey Institute of Neuroscience and Mental Health, The University of Melbourne, Parkville, VIC, Australia; ^2^ School of Chemistry, The University of Melbourne, Parkville, VIC, Australia; ^3^ The Bio21 Institute, The University of Melbourne, Parkville, VIC, Australia

**Keywords:** peptide nucleic acid, cell penetrating peptide, oligonucleotides, native chemical ligation, conjugation

## Abstract

Cell penetrating peptides (CPPs) are being increasingly used as efficient vectors for intracellular delivery of biologically active agents, such as therapeutic antisense oligonucleotides (ASOs). Unfortunately, ASOs have poor cell membrane permeability. The conjugation of ASOs to CPPs have been shown to significantly improve their cellular permeability and therapeutic efficacy. CPPs are often covalently conjugated to ASOs through a variety of chemical linkages. Most of the reported approaches for ligation of CPPs to ASOs relies on methodologies that forms non-native bond due to incompatibility with in-solution phase conjugation. These approaches have low efficiency and poor yields. Therefore, in this study, we have exploited native chemical ligation (NCL) as an efficient strategy for synthesizing CPP-ASO conjugates. A previously characterized CPP [ApoE(133–150)] was used to conjugate to a peptide nucleic acid (PNA) sequence targeting human survival motor neuron-2 (SMN2) mRNA which has been approved by the FDA for the treatment of spinal muscular atrophy. The synthesis of ApoE(133–150)-PNA conjugate using chemo-selective NCL was highly efficient and the conjugate was obtained in high yield. Toward synthesizing trifunctional CPP-ASO conjugates, we subsequently conjugated different functional moieties including a phosphorodiamidate morpholino oligonucleotide (PMO), an additional functional peptide or a fluorescent dye (Cy5) to the thiol that was generated after NCL. The *in vitro* analysis of the bifunctional CPP-PNA and trifunctional CPP-(PMO)-PNA, CPP-(peptide)-PNA and CPP-(Cy5)-PNA showed that all conjugates are cell-permeable and biologically active. Here we demonstrated chemo-selective NCL as a highly efficient and superior conjugation strategy to previously published methods for facile solution-phase synthesis of bi-/trifunctional CPP-ASO conjugates.

## Introduction

CPPs are relatively short cationic, amphipathic peptides (Kurrikoff et al., 2020) and are being widely used for intracellular drug delivery for a wide range of cell-impermeable cargoes, such as therapeutic antisense oligonucleotides (ASOs). The conjugation of CPPs to ASOs has been shown to enhance their cell permeability, intracellular bioavailability, and subsequently, an increase in their therapeutic efficacy (Tajik-Ahmadabad et al., 2017; Klein et al., 2019; Nikan et al., 2020). CPPs have also been utilized to cross the blood-brain barrier for delivery of therapeutic ASOs into the central nervous system (CNS) (Hammond et al., 2016; Shabanpoor et al., 2017). The potential of CPPs as safe and effective delivery vectors is also being recognized and embraced by pharmaceutical industry. Sarepta Therapeutics has shown significant improvement in the pharmacokinetics of PMOs and is developing CPP-PMO conjugates as the next-generation of PMO-based therapy for Duchenne muscular dystrophy (Roberts et al., 2020).

One of the key challenges for developing CPP-ASO conjugates as therapeutics is the large and frequent doses required to achieve the desired pharmacological effect. There is limited evidence of ASO-associated toxicity but CPPs can be toxic even at very low doses. To address this issue, we previously developed trifunctional CPP-ASO conjugates by coupling two ASOs to a single CPP, to mitigate cytotoxicity (Shabanpoor et al., 2015). A key consideration when designing CPP-ASO conjugates is their efficient synthesis. They are often assembled separately via solid-phase synthesis and then conjugated using a variety of chemical linkages, such as disulfide (Turner et al., 2005; Saleh et al., 2010), amide (Bruick et al., 1996; Betts et al., 2012; Shabanpoor and Gait, 2013), thioether (Patil et al., 2019), triazole, oxime, hydrazone, and thiazole bonds (Lu et al., 2010; Nikan et al., 2020). Despite the wide range of ligation reactions available for the preparation of CPP-ASO conjugates, there are still some limitations associated with their use, such as poor *in vivo* stability (e.g., disulfide bonds). Furthermore, linkages with steric bulk impart restricted conformational movement that can potentially affect functional activity. We have previously found that the formation of thiazoles and in particular triazoles (Patil et al., 2019) are slow, and reduce the solubility of the CPP-ASO conjugate.

Ligation reactions that generate native linkages such as amide bonds are more desirable due to their biocompatibility and minimal impact on the physicochemical properties of CPP-ASO conjugates. This method has been routinely used by our group and others to prepare peptide-ASO conjugates (Betts et al., 2012; Shabanpoor and Gait, 2013; Shabanpoor et al., 2015; Klein et al., 2019). The formation of amide linkages is typically achieved via a C-terminal active ester reacting directly with an amine. Unfortunately, this strategy is not compatible with peptide sequences containing lysine, histidine, glutamic acid, and aspartic acid. The aforementioned studies using this conjugation strategy have reported low conjugation yields in the range of 20–40%. Therefore, an improved method for amide bond formation is needed for a more efficient and scalable synthesis of CPP-ASO conjugates.

**SCHEME 1:**
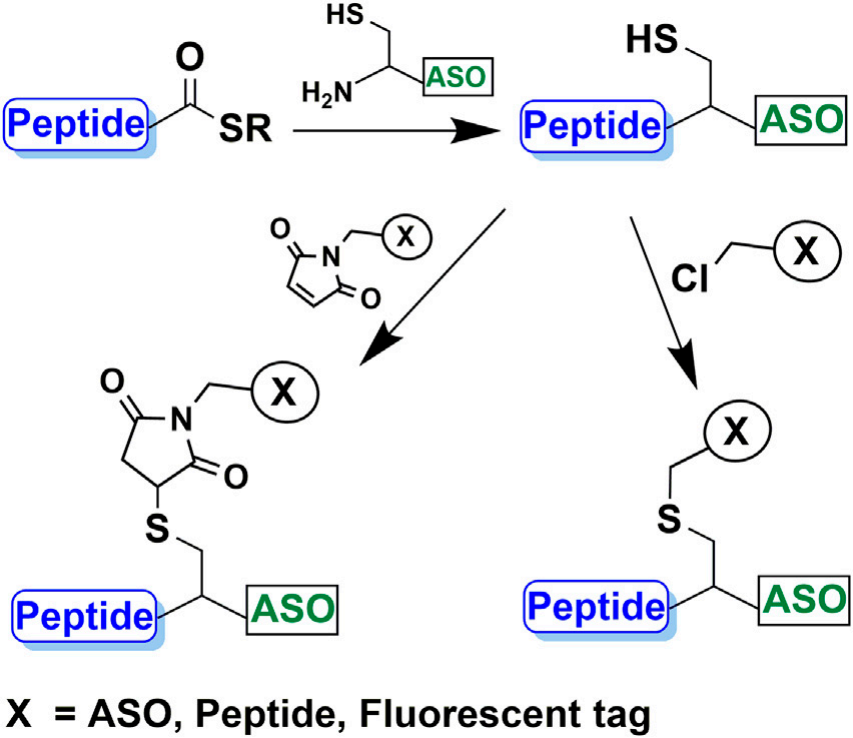
Peptide and ASO conjugation using native chemical ligation and subsequent conjugation of a second moiety such as ASO, peptide or fluorophore to the thiol group.

In this study, we present a simple and efficient chemo-selective method for the preparation of CPP-ASO conjugates, which utilizes NCL to form a stable amide linkage between a CPP and a cysteine-bearing ASO. The highly reactive thiol artifact from the ligation enables a second chemo-selective conjugation with an additional maleimide- or haloacetyl-bearing ASO, bioactive peptide or fluorescent label (Scheme 1). The advantage of using NCL is its tolerability to side-chain functionalities of the unprotected peptides. Besides, it can be performed in mild conditions and does not require special amino acids.

We chose an FDA-approved oligonucleotide sequence (Table 1) targeting survival motor neuron-2 (SMN2) mRNA splice correction as a model ASO (in PNA chemistry) for conjugation to a previously characterized CPP derived from the receptor-binding domain of Apolipoprotein E [ApoE(133–150)] (Meloni et al., 2020). In order to synthesize trifunctional conjugates, three different functional moieties were selected for conjugation to the side-chain of cystine that was generated during NCL of CPP-ASO. These functional moieties include the FDA-approved oligonucleotide sequence as above but incorporated into the PMO scaffold, and a peptide (HA2) derived from the hemagglutinin protein of influenza virus, which enhances endosomal release of conjugated cargoes by disrupting the endosomal membrane (Midoux et al., 1998). It is worth noting that both peptides (ApoE and HA2) either alone and as conjugate to ASOs have been previously characterized by our group and others for cell uptake and also shown to have no significant cytotoxic effect in a concentration range of 20–30 µm (Neundorf et al., 2009; Shabanpoor et al., 2017). The other functional moiety was a fluorophore (Cy5), to visualize the uptake of the CPP-ASO conjugate. To our knowledge, this is the first report on the use of hydrazide-based native chemical ligation for the synthesis of bifunctional CPP-ASO conjugates and subsequent conjugation of a second functional moiety to generate a trifunctional CPP-ASO conjugate.

**TABLE 1:** Sequences, molecular weight and % yield of ASOs, peptides, peptide-PNA conjugate and trifunctional conjugates. Bold indicates where the modifications have been made to different sequences.

PNA/PMO	Sequences	Molecular weight		% Yield
		Exp.	Obt.	
PNA(SMN2)	**Cys**-X-ATTCACTTTCATAATGCTGG-NH_2_	5,656.4	5,656	54
PMO(SMN2)	**Mal**-ATTCACTTTCATAATGCTGG-NH_2_	6,905.4	6,905.8	78
**Peptides**				
ApoE (133–150)-NHNH_2_	Ac-LRVRLASHLRKLRKRLLR-X-G-**NHNH**_**2**_	2,542.4	2,543.4	85
Cl-HA2	**Cl**-X-GLFHAIAHFIHGGWH-NH_2_	1,921.8	1,922.3	79
**Peptide-PNA conjugates**				
C1	Ac-LRVRLASHLRKLRKRLLR-X-G-C-X-ATTCACTTTCATAATGCTGG	8,166.8	8,167.5	74.6
C2	Ac-LRVRLASHLRKLRKRLLR-X-G-C-X-ATTCACTTTCATAATGCTGG **|** Cy5	8,969.8	8,970.7	90
C3	Ac-LRVRLASHLRKLRKRLLR-X-G-C-X-ATTCACTTTCATAATGCTGG **|** Mal-ATTCACTTTCATAATGCTGG	15,071.8	15,072.8	44.6
C4	Ac-LRVRLASHLRKLRKRLLR-X-G-C-X-ATTCACTTTCATAATGCTGG **|** X-GLFHAIAHFIHGGWH-NH_2_	10,052.4	10,053.6	19.2

## Materials and Methods

### Materials

TentaGel^®^ XV resin (0.25 mmol/g) was obtained from Rapp Polymere (Tuebingen, Germany). 2-chlorotrityl chloride polystyrene resin (0.77 mmol/g) was purchased from ChemPep. The 20-mer PMO (5′-ATT​CAC​TTT​CAT​AAT​GCT​GG-3′) was purchased from Gene Tools LLC (Philomath, United States). 9-Fluorenylmethoxycarbonyl (Fmoc) protected L-α-amino acids, 1-[Bis(dimethylamino)methylene]-1H-1,2,3-triazolo [4,5-b]pyridinium-3-oxide hexafluorophosphate (HATU) and aminohexanoic acid (Ahx, X) were purchased from GL Biochem (Shanghai, China). Fmoc-PNA(Bhoc)-OH monomers were purchased from PANAGENE (Daejeon, South Korea). Ethyl cyano (hydroxyimino)acetate (Oxyma Pure) was obtained from Mimotopes (Melbourne, Australia). (1-Cyano-2-ethoxy-2-oxoethylidenaminooxy)dimethylamino-morpholino-carbenium hexafluorophosphate (COMU) was purchased from Chem Impex (United States). Dimethylformamide (DMF), diisoproplyethylamine (DIEA), piperidine and acetonitrile were obtained from Merck (Melbourne, Australia). 8-(9-Fluorenylmethyloxycarbonyl-amino)-3,6-dioxaoctanoic acid (Fmoc-miniPEG-OH) was purchased from IRIS Biotech GMBH (Marktredwitz, Germany). N,N′-Diisopropylcarbodiimide (DIC), triisopropylsilane (TIS), 3,6-dioxa-1,8-octanedithiol (DODT), 4-methylmorpholine (NMP), chloroacetic acid, acetic anhydride, 4-mercaptophenylacetic acid (MPAA), tris(2-carboxyethyl)phosphine hydrochloride (TCEP·HCl) and guanidine hydrochloride (Gn·HCl) were obtained from Sigma-Aldrich (Castle Hill, Australia). Trifluoroacetic acid (TFA) was sourced from Auspep (Melbourne, Australia). SsoAdvanced Universal SYBR Green Supermix and iScript reverse transcription supermix were purchased from BioRad and qPCR primers from Sigma-Aldrich (Melbourne, Australia). Sulfo-cyanine5 maleimide was obtained from Lumiprobe life science solution (Hunt Valley, Maryland). SMA patient-derived fibroblast cells (Type-I, GM03813) were purchased from Coriell Cell Repositories (NJ, United States). µ-Slide 4 Well Ph + Glass Bottom were purchased from ibidi GmbH (Gräfelfing, Germany).

### Peptide Synthesis and Purification

The peptide sequence (HA2): ClAc-X-GLFHAIAHFIHGGWH (X: aminohexanoic acid, ClAc: chloroacetyl) was synthesized as a C-terminal amide on TentaGel XV RAM resin (100–200, 0.25 mmol/g) via Fmoc solid-phase peptide synthesis (SPPS) on a CEM LibertyTM microwave peptide synthesizer. It was synthesized at a 0.1 mmol scale using a 4-fold excess of Fmoc-protected amino acids, which were activated using DIC (4-fold excess) in the presence of OxymaPure (4-fold excess). The removal of Fmoc protecting groups was achieved using piperidine (20% v/v) in DMF. Coupling was carried out once at 90°C for 5 min, except for histidine which was coupled at 50°C for 10 min.

The ApoE peptide hydrazide was assembled manually via Fmoc solid-phase peptide synthesis on a 0.15 mmol scale using 2-chlorotrityl chloride resin as the solid support. After treating 0.3 mmol of resin with a solution of 5% hydrazine and 2% DIEA in DMF, a 50/50 mixture of Fmoc- and Boc-protected glycine was coupled to the resin via COMU/DIEA activation at 50°C (2 eq. of amino acid). This reduced the effective resin loading by approximately 50%, to minimize aggregation on the solid support during peptide chain elongation. The remaining residues were coupled via COMU/DIEA (2 eq.) at 50°C, followed by *N*-terminal acetylation via acetic anhydride/DIEA in DMF.

The resin-bound peptide was cleaved from the solid support by treatment with a cocktail of TFA:DODT:H_2_O:TIPS (94%:2.5%:2.5%:1%), 10 ml for 2 h at room temperature. The ApoE peptide was cleaved without DODT using TFA:H_2_O:TIPS (94%:2.5%:2.5%). Excess TFA was evaporated off and the cleaved peptide was precipitated by addition of ice-cold diethyl ether and centrifuged at 1,500 rpm for 4 min. The peptide pellet was washed in ice-cold diethyl ether twice more. Crude peptides were analyzed and purified by RP-HPLC on Phenomenex Jupiter columns (4.6 × 250 mm, C18, 5 µm) and (21.2 × 250 mm, C18, 10 µm) respectively. 0.1% trifluoroacetic acid in water was used as solvent A and acetonitrile containing 0.1% TFA as solvent B. A gradient of 20–50% B over 30 min was used at a flow rate of 1.5 ml/min for the analytical and 10 ml/min for the preparative column on a WATERS HPLC with a 996 photodiode array detector. Both peptides were purified to greater than 95% purity as determined by analytical RP-HPLC.

### PNA Synthesis and Purification

A 20-mer PNA antisense sequence for human survival motor neuron-2 (*SMN2*) (ATT​CAC​TTT​CAT​AAT​GCT​GG) was synthesized using Fmoc/Bhoc chemistry. The PNA was synthesized on TentaGel XV RAM resin (100–200, 0.25 mmol/g) using the Tribute automated peptide synthesiser at a 20 µmol scale. The Fmoc deprotection was carried out twice using Piperidine 20% in DMF at room temperature for 5 min. The coupling of PNA monomers was achieved using a 4-fold excess of Fmoc-protected PNA(Bhoc)-OH monomers dissolved in NMP activated with HATU (4 eq) and DIEA (8 eq). The coupling was carried out once at room temperature for 1 h. Following final PNA monomer coupling and deprotection, a miniPEG spacer was coupled, followed by incorporation of a cysteine residue at the N-terminus (5′-end). The PNA were cleaved from solid-support by treatment with a cocktail of TFA:H_2_O:TIPS (95:2.5:2.5, v/v/v) for 2 h. The excess TFA was evaporated and the PNAs were precipitated in ice-cold diethyl ether; the PNA pellet was then washed twice with more ether. The crude PNA was analyzed and purifed to greater than 95% purity as described above. The gradient for analysis and purification was 5–35% buffer B over 30 min.

### Conjugation of the Peptide-hydrazide to PNA

The synthesis of the ApoE-PNA conjugate was achieved using native chemical ligation. ApoE-NHNH_2_ (1.5 µmol) was dissolved in 0.2 M sodium phosphate solution containing 6 M Gn.HCl (pH 3.0–3.3). The solution was cooled in an ice-salt bath to at least −15°C. To oxidize hydrazide to azide, NaNO_2_ (10 eq.) was added to the peptide solution on ice and stirred for 15 min. In a separate Eppendorf tube, Cys-PNA (1 µmol) and MPAA (75 µmol) were dissolved in 0.2 M sodium phosphate solution containing 6 M Gn.HCl (pH 3.0–3.3). The reaction mixture was removed from ice and the pH was adjusted to 6.9 with a solution of NaOH (1M). The ligation reaction was monitored by removing 5 µl and quenching it by adding 50 µl of 0.2 M sodium phosphate solution containing 6 M Gn.HCl (pH 3.0–3.3). The reaction was reduced by the addition of 20 µl of 0.1 M TCEP before analyzing with MALDI-TOF and RP-HPLC.

### Synthesis of Trifunctional Peptide-PNA Conjugates

The trifunctional ApoE-PNA conjugates were synthesized by conjugating the maleimide-functionalized sulfo-Cy5 and PMO or chloroacetylated HA2 (Cl-HA2) to the thiol of cysteine generated during native chemical ligation of ApoE to PNA. ApoE-PNA and Maleimide-Sulfo-Cy5, Maleimide-PMO or Cl-HA2 (1.5 eq) were dissolved in 0.1 M phosphate buffer (pH 7.5). The two solutions were mixed and the pH readjusted to 7.5 with NH_4_HCO_3_, to offset the presence of TFA counterions. The reaction was monitored by MALDI-TOF mass spectrometry and the absence of peptide-PNA indicated completion of reaction. In the case of Cl-HA2, due to the lack of conjugate formation between the peptide-PNA and Cl-HA2, the pH of the reaction was increased, but unfortunately a precipitate started to appear, despite the addition of acetonitrile and heating. However, once the pH was lowered to <7, the precipitate disappeared, and the reaction solution was clear. The formation of the trifunctional ApoE-(HA2)-PNA conjugate was monitored with MALDI-TOF and a significant amount of unreacted ApoE-PNA and Cl-HA2 was observed. This was confirmed with HPLC analysis. As the reaction between chloroacetyl and thiol groups require a pH of >7.5 and Cl-HA2 is poorly soluble at this pH, the resultant yield was low for this conjugate. The concentration of the peptide-PNA and trifunctional conjugates was determined by measuring the molar absorption of the conjugates at 265 nm in 0.1 N HCl solution.

### Mass Spectral Characterization of Peptides and Peptide-PNA Conjugates

Matrix-assisted laser desorption ionization time-of-flight mass spectrometry (MALDI-8020, Shimadzu) was used to characterize the peptides and peptide-PNA conjugates with sinapinic acid as the matrix.

### Cell Culture, Transfection and Confocal Imaging

Spinal muscular atrophy (SMA)-patient-derived fibroblasts were used to test the antisense activity and Cy5-fluorescent uptake of the peptide-PNA and trifunctional peptide-PNA conjugates. The cells were maintained in DMEM with 10% fetal bovine serum, 1% L-glutamine and 1% penicillin-streptomycin at 37°C with 5% CO_2_. Fibroblasts were plated at a density of 3 × 10^5^ cells per 2 ml per well in 6 well plates. The peptide-PNA and trifunctional peptide-PNA conjugate concentrations (1 and 2 µm) were made up in serum-free Opti-MEM and added to each well and incubated for 4 h at 37°C. The transfection medium was then replaced with growth medium and cells incubated for a further 20 h at 37°C. Cells were washed with PBS once and stored at −80°C until ready for RNA extraction.

For fluorescent uptake studies, SMA fibroblast cells were plated at a density of 60,000 cells/well of a µ-Slide 4 Well ^Ph+^ Glass Bottom chamber slides precoated with poly-L-ornithine. The cells reached 80% confluency after 24 h. They were treated with Cy5-labelled peptide-PNA conjugates made up at a concentration of 1 and 2 µm in OptiMEM without serum for 1 h at 37°C. At 15 min to the end of incubation time, Hoechst (1:1,000 dilution) was added to each well. Cells were washed with PBS and images of cells were taken using the Leica SP8 Resonant Scanning microscope with 63×/1.4 oil objective. The images were acquired at the Cy5-specific excitation of 633 nm and emission of 638–779 nm and Hoechst excitation and emission of 405 nm and 410–585 nm, respectively.

### RNA Extraction and RT-qPCR Analysis

Total RNA was extracted using the ISOLATE II RNA Mini Kit (Bioline, Australia) as per the manufacturer’s protocol. The purified RNA (400 ng) was subsequently reverse transcribed to single-stranded complementary DNA (cDNA). The transcription was performed using the Veriti Thermal Cycler (ThermoFisher Scientific) following the thermal cycling conditions: 5 min at 25°C for priming, 20 min at 46°C for reverse transcription and finally 1 min at 95°C for reverse transcription inactivation, as stated in the manufacturer’s protocol (BioRad, Australia).

Quantitative PCR was subsequently carried out using 20 ng of cDNA per well of 96-well plate in triplicates for each treatment, using 2X Fast SYBR Green Mastermix (ThermoFisher Scientific). The CFX96 Touch Real-Time PCR Detection Cycler, from BioRad, was used to perform the qPCR under the following conditions: 95°C for 2 min, followed by 39 cycles of amplifications, with 95 °C for 5 s and 60°C for 30 s, then 95°C for 5 s and finally to determine the melt curve, the temperature was increased from 65°C to 95°C using increments of 0.5°C. The amplification was carried out using *SMN2*-specific primers (Fw:5′-GCTTTGGGAAGTATGTTAATTTCA-3′, Rv: 5′-CTA​TGC​CAG​CAT​TTC​TCC​TTA​ATT-3′) for detecting full-length *SMN2* mRNA transcripts and the human *HPRT1* (Fw: 5′-GAC​CAG​TCA​ACA​GGG​GAC​AT-3′, Rv: 5′-CCT​GAC​CAA​GGA​AAG​CAA​AG-3′) as the reference gene. The cycle thresholds (Ct) of all triplicates were averaged and analyzed using the ∆∆Ct method, corrected against the house-keeping gene’s Ct values. The resulting values were further normalized to the untreated control values, which were set to 1. Data were analyzed using GraphPad Prism (v 8.4.3, San Diego, CA, United States) and expressed as mean ± standard error of the mean (SEM) from at least three independent experiments. The statistical significance of the data was determined using One-way ANOVA with post-hoc Bonferroni and a *p* value of <0.05 was considered statistically significant.

## Results and Discussion

The PNA was synthesized using previously established Fmoc/Bhoc solid-phase synthesis protocols (Tailhades et al., 2017) and functionalized with cysteine at the N-terminus (5′-end). A polyethylene glycol spacer (miniPEG) was introduced between the PNA and the cysteine. Cys-miniPEG-PNA was purified via reversed-phase HPLC and obtained in an excellent yield (54%) (Table.1, ESI Supplementary Figures S1C, S2C). The CPP [ApoE (133–150)] was synthesized as a C-terminal hydrazide and a miniPEG spacer was placed between the C-terminal glycyl hydrazide and the peptide sequence. The HA2 peptide was N-chloroacetylated to enable conjugation to the thiol side-chain of cysteine in the peptide-PNA conjugate. Both peptides were purified to greater than 95% purity as determined by analytical HPLC and obtained in a high yield (Table 1, ESI Supplementary Figures S1, S2). In order to synthesize the trifunctional peptide-(PMO)-PNA conjugate, a 20-mer PMO with the same sequence as the PNA (Table 1) was functionalized at its 3′-end with maleimidopropionic acid, as previously described (Tajik-Ahmadabad et al., 2017). The purified Maleimide-PMO was obtained in excellent yield (78%) (ESI Supplementary Figures S1D, S2D).

Conjugation of Cys-PNA with the peptide hydrazide was carried out by *in situ* oxidation of the C-terminal hydrazide with NaNO_2_ to the corresponding acyl azide, followed by thiolysis to obtain the required thioester (Fang et al., 2011; Zheng et al., 2013). There are three other reported methods describing the conjugation of peptides to ASOs via NCL (Stetsenko and Gait, 2000; Stetsenko and Gait, 2001; Diezmann et al., 2010; Jang et al., 2020). The first published method requires isolation of a thiol-functionalized ASO and a peptide N-terminally capped with a thioester (Stetsenko and Gait, 2000; Stetsenko and Gait 2001). This approach, however, requires the preparation of specialized precursors and/or additional deprotection steps. Isolation of peptide thioesters can sometimes be challenging due to the risk of hydrolysis during subsequent handling and purification. The other two approaches rely on modified reactive nucleobases such as Oxanine for incorporation into the ASO sequence during solid-phase assembly. A drawback of such approaches is opening of the nucelobase ring structure during ligation, which can potentially cause steric hindrance due to its close proximity to the thiol group (Jang et al., 2020). However, the *in-situ* approach used in this study, does not require any modifications or special reactive nucleobases such as Oxanine.

**FIGURE 1:**
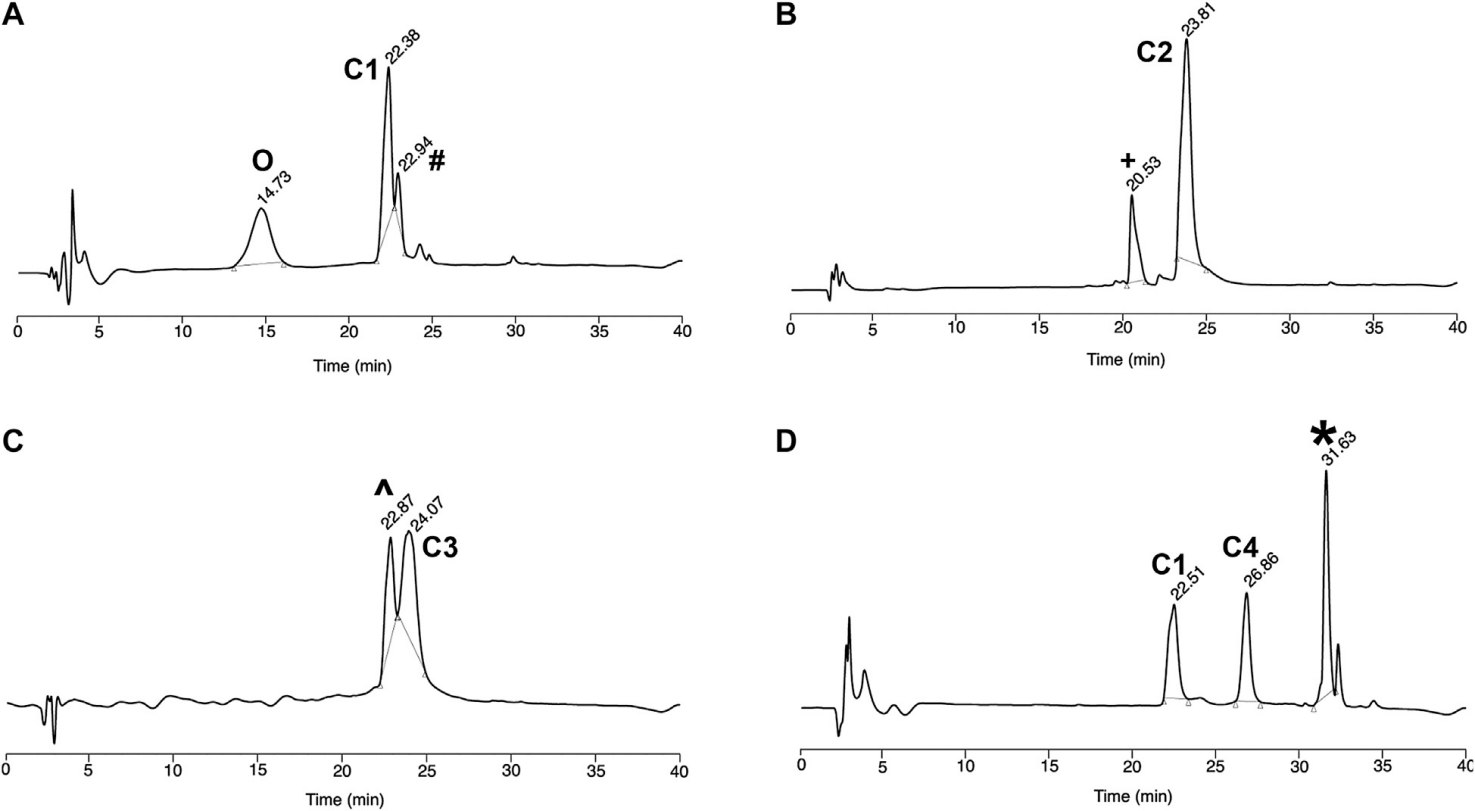
Reverse-Phase HPLC analysis of **(A)** CPP-PNA conjugate (C1) formed using native chemical ligation **(B–D)** trifunctional conjugates C2, C3, and C4 respectively. O PNA without N-terminal Cysteine, # Unreacted peptide thioester, + Malimide-Sulfo-Cy5, ^ Mix of C1 conjugate and modified maleimide-PMO, *Unreacted Cl-HA2 peptide.

First, we synthesized the CPP-PNA conjugate (C1) by treating the CPP hydrazide with NaNO2 at pH 3.3 in a −15°C ice/salt bath for 20 min 4-mercaptophenylacetic acid (MPAA), followed by Cys-PNA, were then added to the reaction mixture and the pH was adjusted to 6.9. The reaction was completed in 30 min, as indicated by the disappearance of the signal corresponding to the Cys-PNA species in the MALDI-TOF and RP-HPLC spectra (Figure 1A). PNA with a cysteine deletion co-eluted during purification but nevertheless, the CPP-PNA conjugate (C1) was isolated and obtained in good yield (75%) based on the mass of starting Cys-PNA (ESI Figure 2A, Supplementary Figures S3A, S4A, Table.1). This is significantly higher than yields of 10–60% obtained using previously reported approaches for the solution-phase conjugation of peptide-oligonucleotides via amide bond formation (Diezmann et al., 2010; Shabanpoor and Gait, 2013).

**FIGURE 2:**
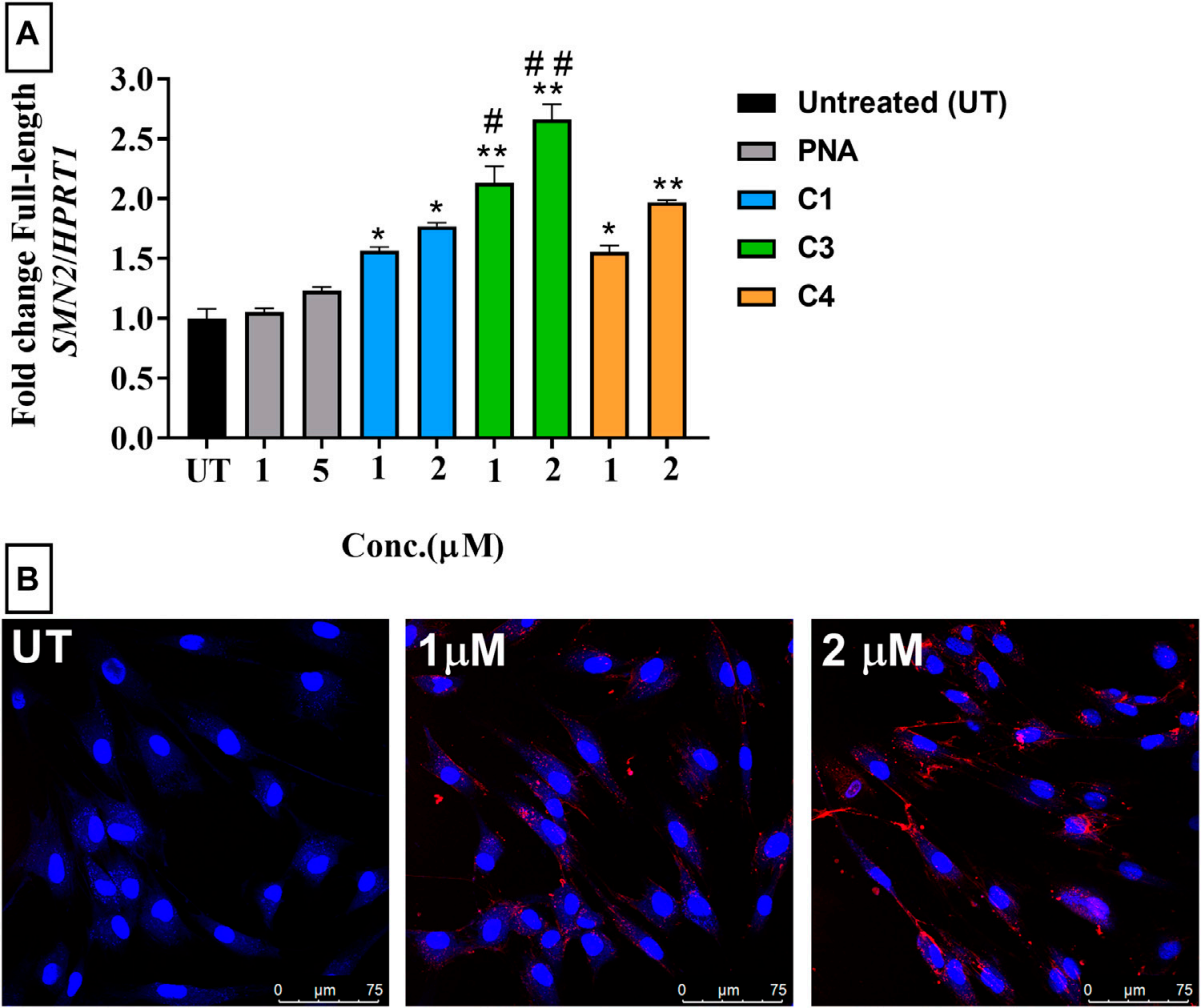
**(A)** RT-qPCR analysis of antisense activity (change in the level of full-length SMN2 mRNA) in SMA patient-derived fibroblasts treated with PNA (1 and 5 µm) and ApoE-PNA conjugate (C1-blue) and trifunctional conjugates C3 (green) and C4 (orange) (1 and 2 µm). N = 3, *p < 0.01, **p < 0.001 cf. untreated and PNA. #*p* < 0.05 cf. 1 µm C1, ##*p* < 0.001 cf. 2 µm C1. **(B)** Confocal microscopy images of fibroblasts treated with Cy5-labelled ApoE-PNA conjugate (C2) at 1 µm and 2 µm for 1 h at 37°C. Scale bar: 75 µm.

**FIGURE 3:**
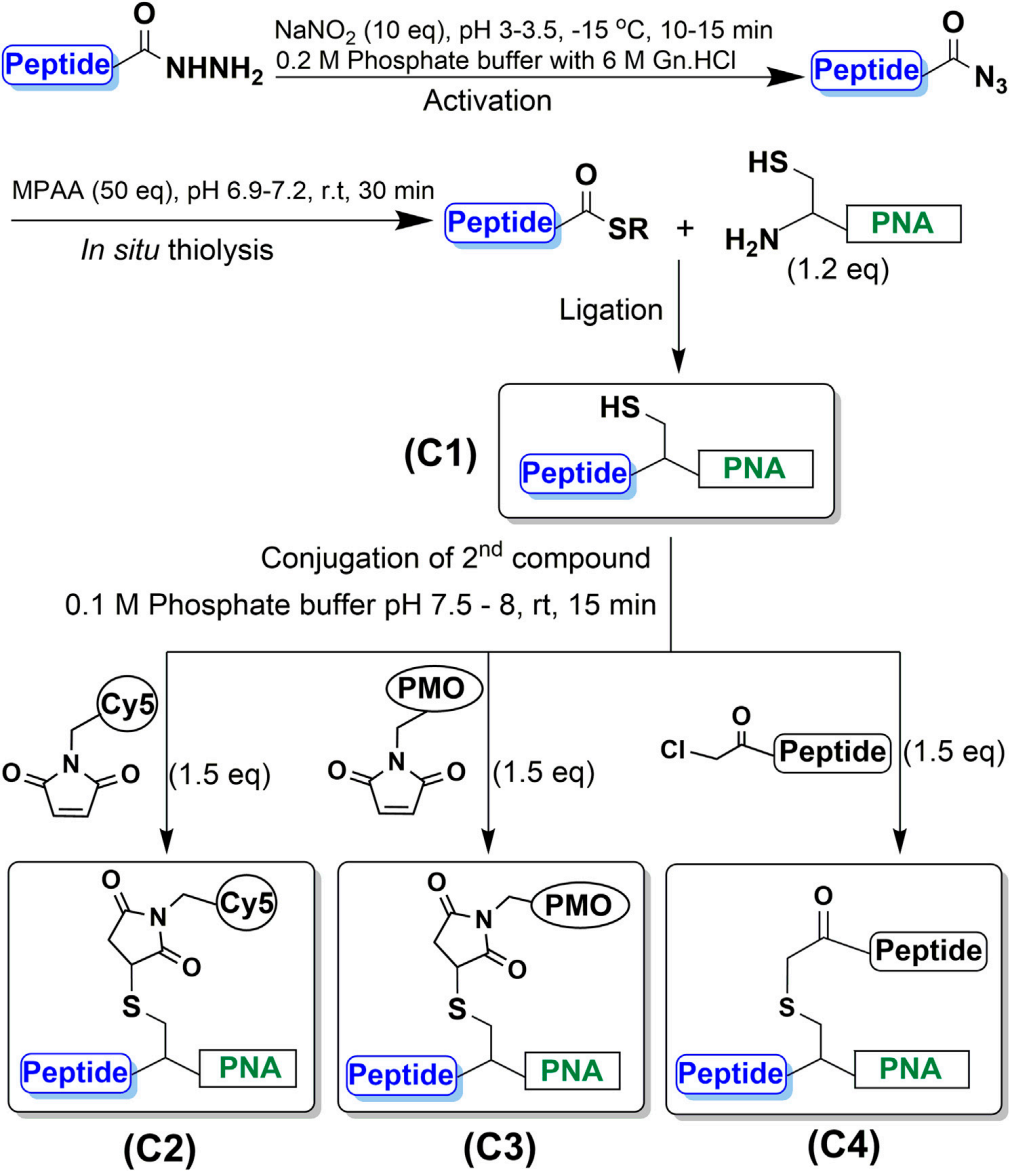
Synthesis of CPP-PNA conjugate (C1) using native chemical ligation. Initial step of peptide-hydrazide activation using NaNO_2_ (10 eq) at pH 3–3.5 and −15°C for 20 min followed by *in situ* thiolysis to generate thioester using MPAA (50 eq) which reacts with the N-terminal cysteine to form an amide linkage. Subsequent synthesis of trifunctional conjugates (C2, C3, C4) by coupling a variety of second functional moieties to the side-chain of cysteine through thioether linkages via thiol-halide SN2 and thiol-maleimide Michael addition reactions using 1.5-fold excess of maleimide- and haloacetyl-functionalized moieties at pH 7.5–8.

Subsequently, the CPP-PNA conjugate was further functionalized via the free thiol. The first trifunctional conjugate (C2) was prepared by forming a thioether between the free thiol group and a maleimide-functionalized fluorophore, Cy5, which was complete in 15 min (Figure 1B). This trifunctional conjugate was obtained in a high yield of 90% based on the amount of starting CPP-PNA material (C1) (Supplementary Figures S3B, S4B). The formation of the trifunctional conjugate C3 (CPP-(PMO)-PNA) (ESI Supplementary Figures S3C, S4C, Table 1) was also achieved using the thiol-maleimide “click” reaction to form a thioether linkage. Progress of the reaction was analyzed at 15 min (Figure 1C). MALDI-TOF analysis of the two peaks showed the peak eluting at 24.1 min to be the desired trifunctional conjugate. The peak at 22.9 min showed a mixture of unreacted CPP-PNA and an unidentified maleimide-PMO adduct (+80 m/z). An isolated yield of 45% was obtained for the C3 conjugate. The synthesis of trifunctional conjugate C4 (Figure 3) was achieved using the thiol-halide SN2 reaction instead of the thiol-maleimide Michael addition. The HA2 peptide was functionalized at the N-terminus with chloroacetyl via the corresponding carboxylic acid and carbodiimide activation. The SN2 reaction was initially performed at pH 7.5, but the expected product was not observed, therefore, the pH of the reaction mixture was increased to 8. This resulted in formation of desired trifunctional C4 conjugate (Figure 1D). However, we observed precipitate formation in the reaction mixture, due to insolubility of the HA2 peptide at the elevated pH, which is close to its iso-electric point. The poor solubility of HA2 under these reaction conditions led to a lower than expected yield (19.2%). This is intrinsic to this peptide and we believe that peptides with good solubility at pH 7-8 will have higher conjugation efficiency and yields. Nevertheless, as proof-of-concept, we have shown that the thiol group that is generated during NCL of CPP-PNA conjugates can be utilized to ligate a variety of functional moieties, to generate trifunctional CPP-ASO conjugates.

The activity of the peptide-PNA and trifunctional conjugates were evaluated in an SMN2 exon-inclusion assay using spinal muscular atrophy (SMA) patient fibroblasts. This assay measures the level of full-length SMN2 mRNA which indicates the efficacy of the peptide (ApoE) in delivering the conjugated PNA and PMO into the cells. The conjugates were tested at 1 and 2 µm concentrations to enable comparison of CPP efficiency in delivering the conjugated PNA, PMO and fluorophore into the cells. The maximum concentration for CPP-ASO conjugates was set at 2 µm, as higher concentrations would results in saturation of response based on our previous studies on cellular uptake of ApoE and similar peptides (Shabanpoor et al., 2017; Tajik-Ahmadabad et al., 2017). All conjugates significantly increased the level of full-length SMN2 compared to untreated cells, as measured by RT-qPCR (Figure 2A). The trifunctional C3 conjugate showed higher activity compared to the peptide-PNA (C1) conjugate due to the higher amount of ASO being delivered into the cells coupled to a single CPP. Confocal microscopy imaging of the Cy5-labelled trifunctional (C2) conjugate showed a concentration-dependent increase in uptake in fibroblasts (Figure 2B). The higher level of C2 uptake at higher concentration correlates with the level of activity obtained for C1, which is the unlabelled analogue of C2. Given that the trifunctional C4 conjugate has an endosomal disrupting peptide, HA2, it was expected to have higher antisense efficacy compared to the peptide-PNA conjugate (C1). Although C4 showed a concentration dependent increase in the activity, which is higher than PNA alone, the antisense activity is not statistically significant compared with activity of C1. This can be due to the partial solubility of the C4 in the cell culture media which prevents its efficient cellular uptake.

## Conclusion

In summary, we have demonstrated for the first time the use of hydrazide-based NCL as an efficient and site-specific approach for the preparation of trifunctional peptide-ASO conjugates. This methodology utilizes the thiol artifact from the NCL as an additional biorthogonal handle for further functionalization. We have demonstrated that incorporation of a second functional moiety does not hamper CPP-PNA uptake or activity. Here we have also shown that two ASO sequences conjugated to a single CPP can be simultaneously delivered into cells. In principle, this method is applicable to the preparation of any trifunctional CPP-ASO conjugate.

## Data Availability

The original contributions presented in the study are included in the article/Supplementary Material, further inquiries can be directed to the corresponding author.
