# Supplementary material for: Modular Synthesis of Trifunctional Peptide-oligonucleotide Conjugates via Native Chemical Ligation
Source: Front Chem. 2021 Mar 2;9:627329. doi: 10.3389/fchem.2021.627329 (PMC7962911; doi:10.3389/fchem.2021.627329)
Supplement: Supplementary file 1 [file datasheet1.docx]

Supplementary Material

# Modular synthesis of trifunctional peptide-oligonucleotide conjugates via native chemical ligation

D. Dastpeyman, J.A. Karas, A. Amin, B.J. Turner, and F. Shabanpoor*

*** Address correspondence to:** [fazel.shabanpoor@unimelb.edu.au](mailto:fazel.shabanpoor@unimelb.edu.au)


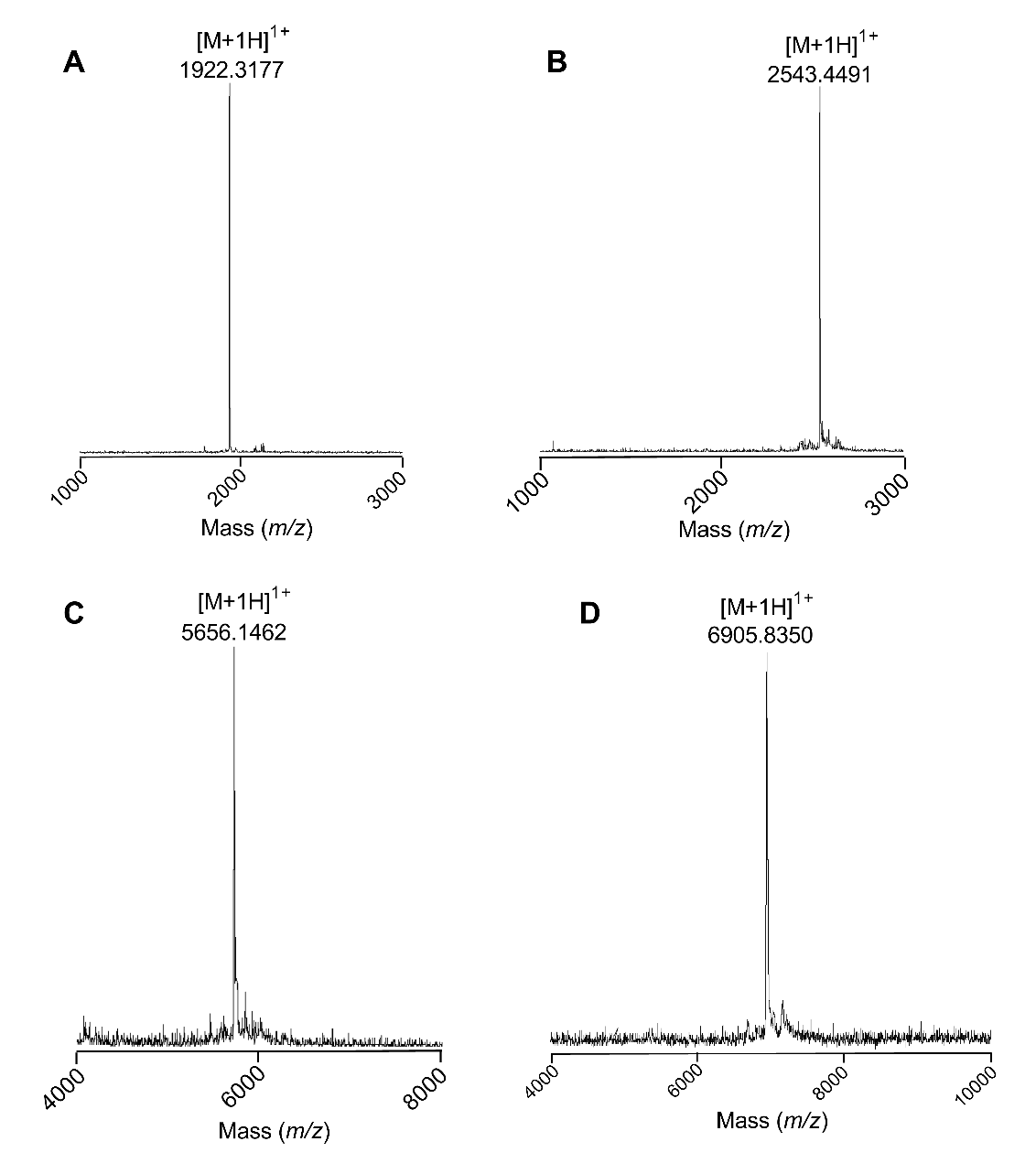


Fig. S1 MALDI-TOF mass spectra of (A) N-chloroacetylated HA2 peptide (B) ApoE(133-150) peptide with C-terminal hydrazide (C) Cys-miniPEG-PNA and (D) 3’-Malimide-PMO.


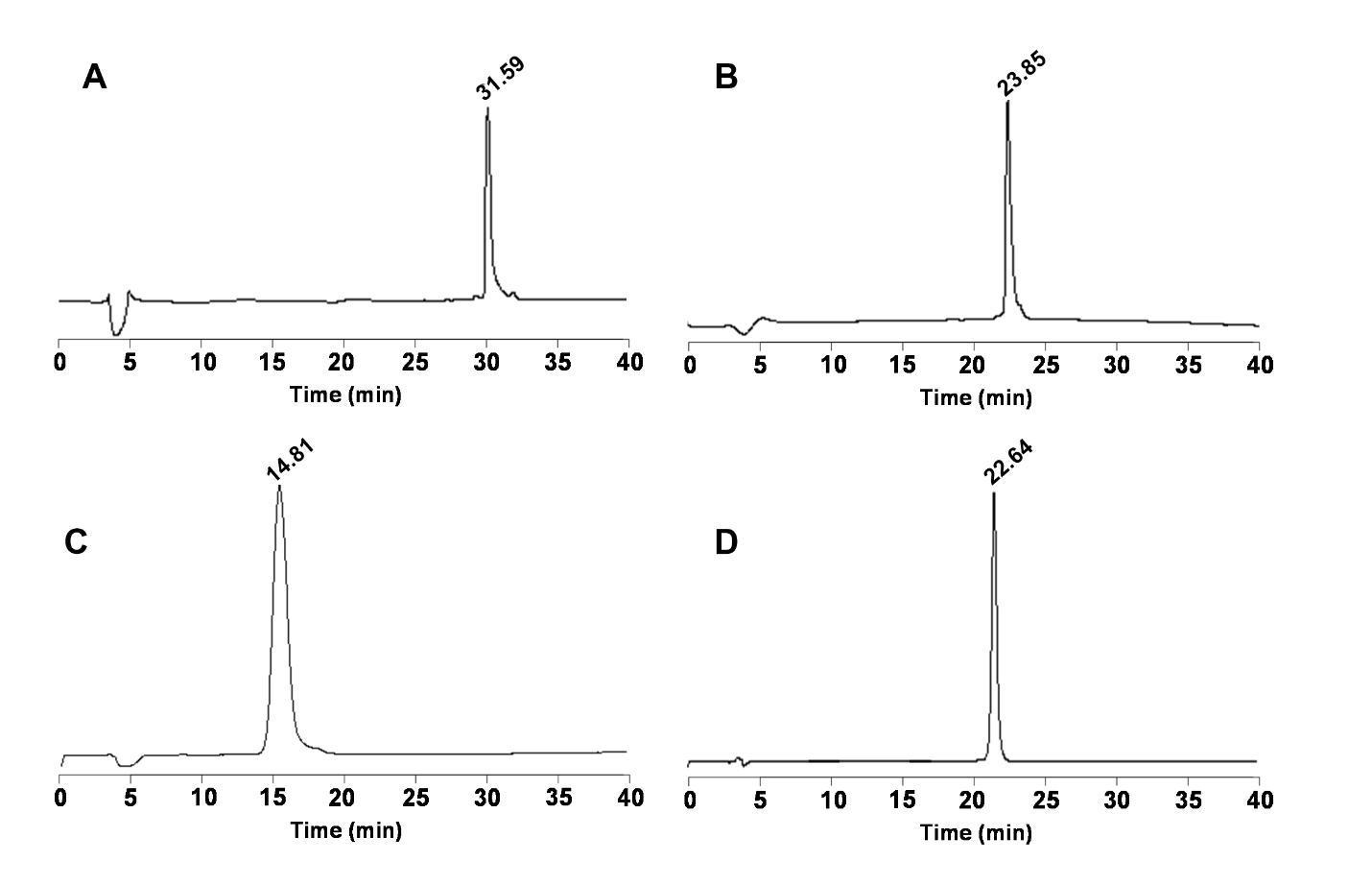


**Fig. S2** HPLC traces of purified (**A**) N-chloroacetylated HA2 peptide (**B**) ApoE(133-150) peptide with C-terminal hydrazide (**C**) Cys-miniPEG-PNA and (**D**) 3’-Malimide-PMO.


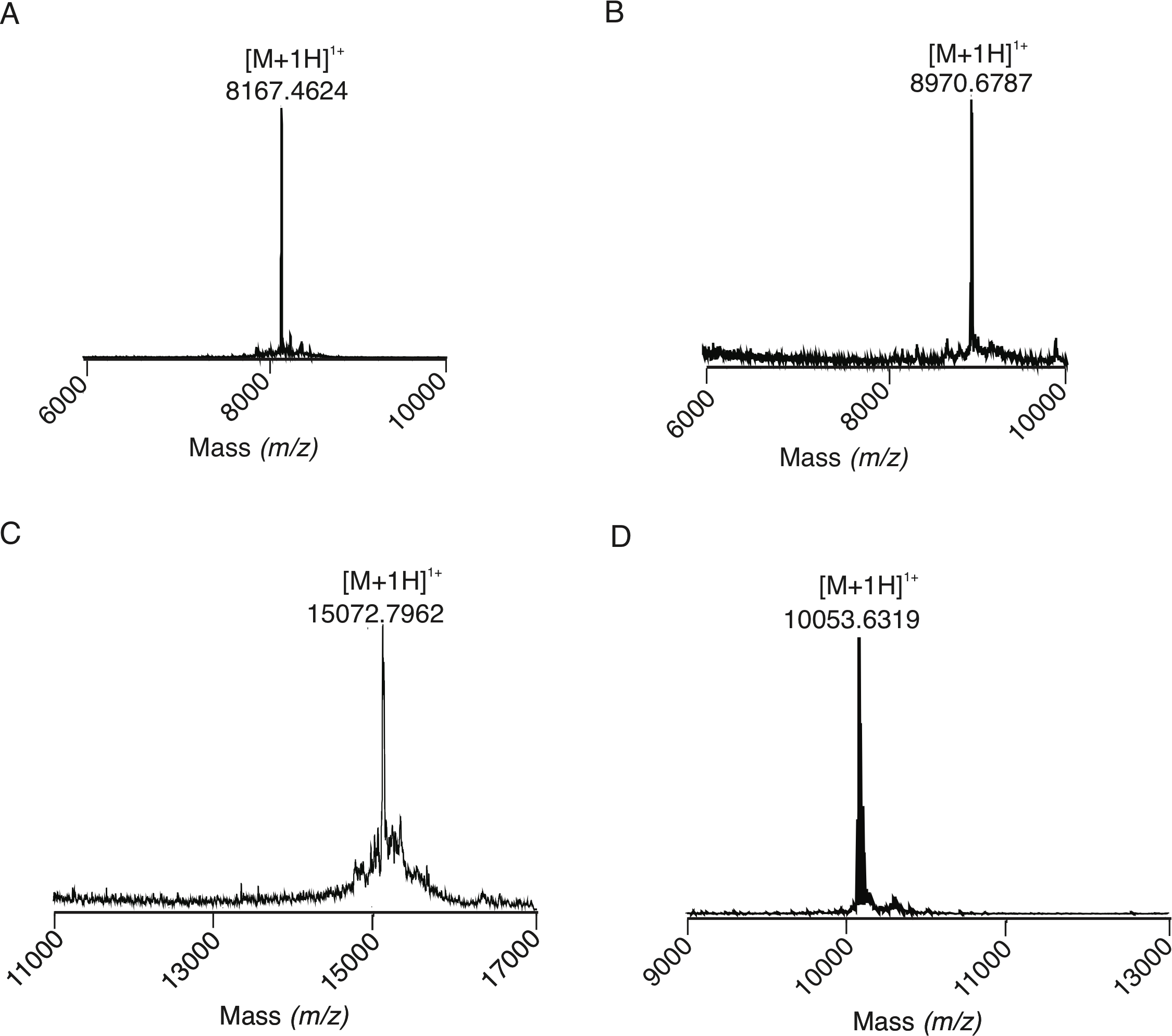


Fig. S3 MALDI-TOF mass spectra of (A) ApoE-PNA conjugate and trifunctional conjugate (B) ApoE-(Sulfo-Cy5)-PNA, (C) ApoE-(PMO)-PNA and (D) ApoE-(HA2)-PNA.


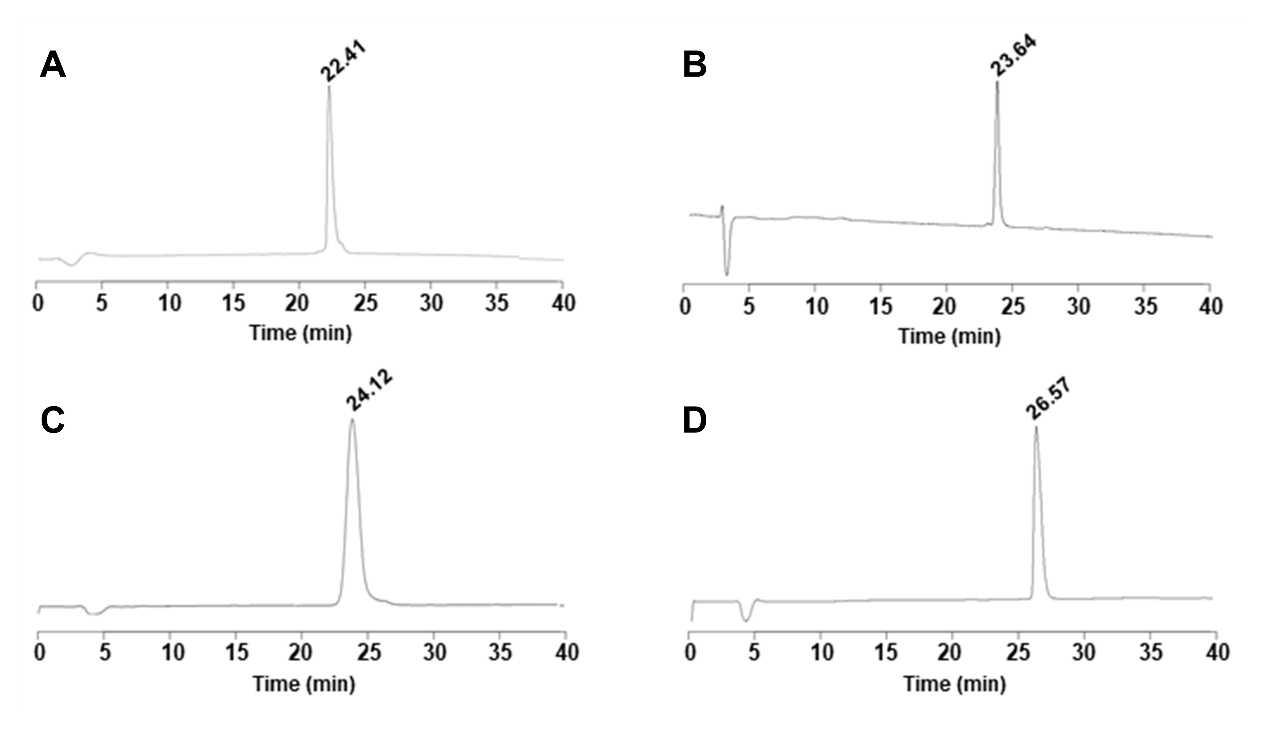
 Fig. S4 HPLC traces of purified of (A) ApoE-PNA conjugate and trifunctional conjugate (B) ApoE-(Sulfo-Cy5)-PNA, (C) ApoE-(PMO)-PNA and (D) ApoE-(HA2)-PNA.
